# Microdroplet fusion mass spectrometry: accelerated kinetics of acid-induced chlorophyll demetallation

**DOI:** 10.1017/S0033583517000014

**Published:** 2017-01

**Authors:** Jae Kyoo Lee, Hong Gil Nam, Richard N. Zare

**Affiliations:** 1 Department of Chemistry, Stanford University, Stanford, California 94305, USA; 2 Center for Plant Aging Research, Institute for Basic Science (IBS) and Department of New Biology, DGIST, Daegu 711-873, Republic of Korea

## Abstract

Kinetics of acid-induced chlorophyll demetallation was recorded in microdroplets by fusing a stream of microdroplets containing 40 μM chlorophyll *a* or *b* dissolved in methanol with a stream of aqueous microdroplets containing 35 mM hydrochloric acid (pH = 1·46). The kinetics of the demetallation of chlorophyll in the fused microdroplets (14 ± 6 μm diameter; 84 ± 18 m s^−1^ velocity) was recorded by controlling the traveling distance of the fused microdroplets between the fusion region and the inlet of a mass spectrometer. The rate of acid-induced chlorophyll demetallation was about 960 ± 120 times faster in the charged microdroplets compared with that reported in bulk solution. If no voltage was applied to the sprayed microdroplets, then the acceleration factor was about 580 ± 90, suggesting that the applied voltage is not a major factor determining the acceleration. Chlorophyll *a* was more rapidly demetallated than chlorophyll b by a factor of ~26 in bulk solution and ~5 in charged microdroplets. The demetallation kinetics was second order in the H^+^ concentration, but the acceleration factor of microdroplets compared with bulk solution appeared to be unchanged in going from pH = 1·3 to 7·0. The water:methanol ratio of the fused microdroplets was varied from 7:3 to 3:7 causing an increase in the reaction rate of chlorophyll *a* demetallation by 20%. This observation demonstrates that the solvent composition, which has different evaporation rates, does not significantly affect the acceleration. We believe that a major portion of the acceleration can be attributed to confinement effects involving surface reactions rather than either to evaporation of solvents or to the introduction of charges to the microdroplets.

## 1. Introduction

Chlorophyll is a pigment molecule that plays a central role in photosynthesis by absorbing and transferring solar energy (Hynninen & Leppäkases, 2004). Demetallation of core Mg^2+^ or Zn^2+^ from chlorophylls is an important process for the production of electron acceptors in photosynthesis and degradation pathways of chlorophylls (Saga & Tamiaki, 2012). In bulk solution, the kinetics of the acid-induced chlorophyll demetallation was characterized by Tamiaki, Saga and co-workers (Hirai *et al.* 2009; Saga *et al.* 2012; Saga & Tamiaki, 2012) who determined that the kinetics was second order in the H^+^ concentration, but first order in the chlorophyll concentration. This result is expected when the central Mg^2+^ or Zn^2+^ ion is replaced by two H^+^ ions.

We have previously reported that some chemical reaction rates are markedly accelerated in charged microdroplets compared with bulk solution (Banerjee & Zare, 2015; Lee *et al*. 2015a, b). Similar observations have been made by other groups (Badu-Tawiah *et al.* 2012; Bain *et al.* 2015; Fallah-Araghi *et al.* 2014; Girod *et al.* 2011; Müller *et al.* 2012). We investigate the kinetics of chlorophyll demetallation in microdroplets to seek an understanding of the mechanism of the reaction acceleration in the confined microenvironment of a microdroplet.

We have developed a new mass spectrometric technique for measuring liquid-phase kinetics in microdroplets, which we name microdroplet fusion mass spectrometry. This technique has three advantages: it allows: (1) recording fast chemical reactions on the microsecond timescale; (2) capturing early molecular events occurring within a few microseconds; and (3) studying liquid-phase kinetics in microdroplets, which can differ markedly from that found in bulk solution. Microdroplet fusion mass spectrometry was employed to study chlorophyll demetallation kinetics. We have examined the effect of applied voltage in the generation of electrosprayed charged microdroplets on the chlorophyll demetallation rate. We investigated chlorophyll *a* and *b* to learn how different side chains affect reaction rate acceleration. The effect of different methanol–water solvent compositions on the reaction rate acceleration was also examined. We find that the microdroplet chemistry is primarily governed by surface reaction and that the extremely large surface-to-volume ratio of microdroplets compared with bulk solution contributes significantly to the observed acceleration of the reaction rate in microdroplets.

## 2. Materials and methods

### 2.1. Chemicals and sample preparation

Chlorophyll *a* and *b* were purchased from Sigma (St. Louis, MO, USA). HPLC grade methanol, hydrochloric acid and water were purchased from Fisher Scientific (Nepean, ON, Canada).

### 2.2. Examination of chlorophyll demetallation in bulk solution

Stock solutions of chlorophyll *a* and *b* were prepared at 1 mM in methanol. Working solutions of chlorophylls at 40 μM were prepared by diluting the stock solution in methanol. HCl solution prepared at 70 mM concentration in water was mixed with the chlorophyll solutions at 1:1 (v/v) ratio with the final HCl concentration of 35 mM. The mixed solutions were gently stirred and incubated at room temperature for 20 min. Mass spectrometric analyses of the mixed solutions were carried out to determine the identities of the species present using an Orbitrap mass spectrometer (Thermo Scientific LTQ Orbitrap XL Hybrid Ion Trap-Orbitrap; Thermo Scientific, MA, USA).

### 2.3. Microdroplet fusion mass spectrometry for chlorophyll kinetics

The kinetics analysis using microdroplet fusion mass spectrometry was conducted as previously described (Lee *et al.* 2015b). Briefly, Thermo Scientific LTQ Orbitrap XL Hybrid Ion Trap-Orbitrap mass spectrometer was used for the kinetic studies of chlorophyll demetallation in fused microdroplets in positive ion mode. A high-pressure dried N_2_ gas at 120 psi was used to generate a stream of microdroplets. The voltage applied to the syringes of the two droplet sources was varied between 0 and +5 kV to examine the effect of applied voltage on the kinetics of chlorophyll demetallation. The two microdroplet sources are equipped with an X–Y–Z micropositioning linear and angular stage for accurate alignment of the two microdroplet streams. This alignment is important for ensuring fusion of most of the incident droplets and to maintain a linear trajectory toward the mass spectrometer inlet. The best alignment was acquired with the angle between two crossed microdroplet streams at 78°, which showed the highest probability of droplet fusion and straight trajectories of the fused microdroplets to the inlet of the mass spectrometer. Two solutions of analytes (chlorophyll *a* or *b* at 40 μM and HCl at different concentrations ranging between 0 and 100 mM) were injected from the two microdroplet sources with a syringe pump (Harvard Apparatus, Holliston, MA, USA) at a flow rate of 30 μl min^−1^. The heated capillary temperature was maintained at approximately 275 °C. The capillary and tube lens voltages were set as 13 and 190 V.

## 3. Results

### 3.1. Kinetics of chlorophyll demetallation in microdroplets

**Fig. 1:**
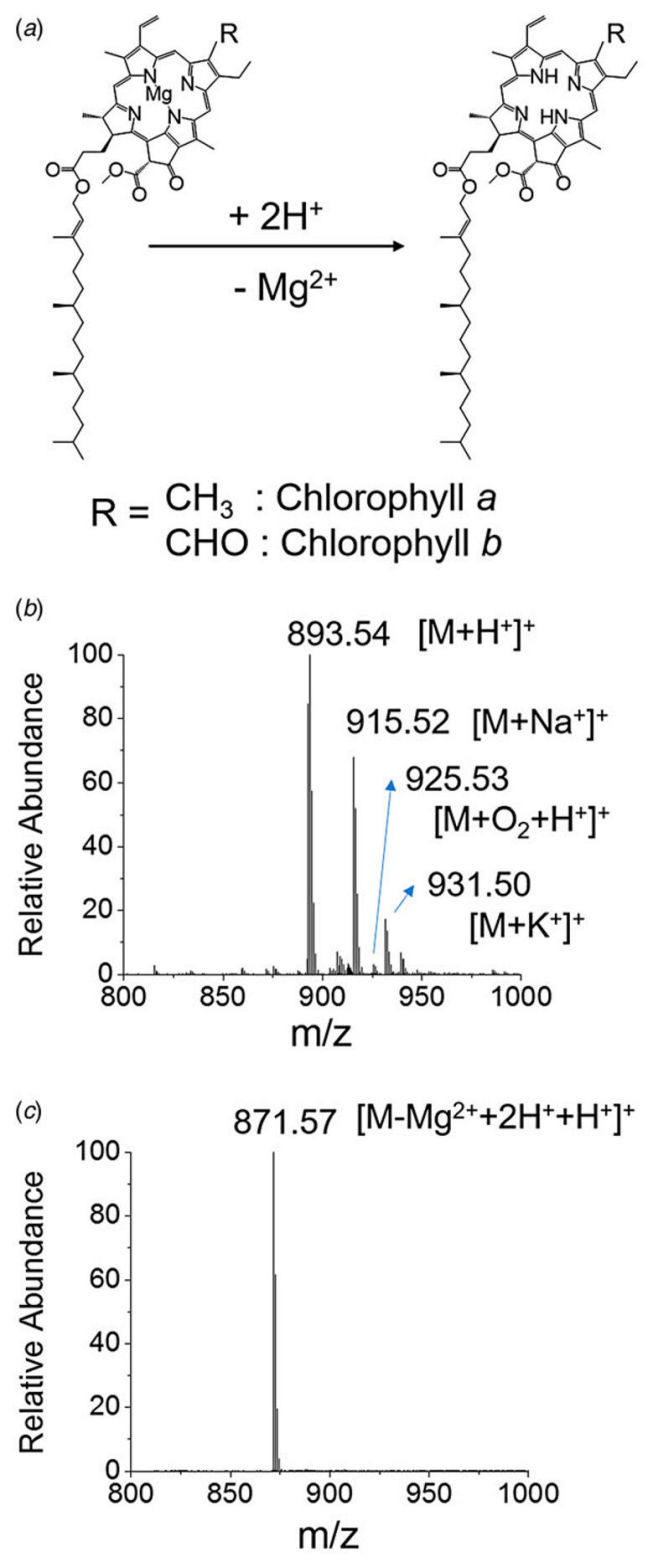
(*a*) Reaction scheme of acid-induced demetallation of chlorophyll to form phaeophytin. Mass spectra of chlorophyll *a*, (*b*) before and (*c*) after adding 70 mM HCl.

Figure 1*a* presents the acid-induced demetallation of chlorophyll *a* to form phaeophytin. The central Mg^2+^ ion in chlorophyll *a* is substituted by two hydrogen ions at high hydrogen ion concentration. ESI mass spectra of chlorophyll *a* before and after adding 35 mM HCl are shown in Supplement Figs 1*b* and 1*c*. The chlorophyll *a* without treating with HCl exhibited a protonated mass peak ([M + H^+^]^+^) at *m*/*z* 893·53 with a sodiated peak ([M + Na^+^]^+^) at *m*/*z* 915·52. The chlorophyll *a* with potassium adduct ([M + K^+^]^+^) was observed at *m*/*z* 931·50. An oxidized species at *m*/*z* 925·53 was detected in a relatively low intensity. Chlorophyll *a* after treating for 20 min with 35 mM HCl exhibited a protonated phaeophytin mass peak ([M−Mg^2+^ + 2H ^+^ +H^+^]^+^) at *m*/*z* 871·57. Negligible molecular species with sodium and potassium adducts were detected in the demetallated chlorophyll *a* with HCl treatment. The high hydrogen ion concentration drove a major portion of formed ions to the protonated species. It is to be noted that oxidized peaks observed in intact chlorophyll *a* also disappeared in demetallated chlorophyll *a*, suggesting that acid-induced demetallation interferes with oxidation.

**Fig. 2:**
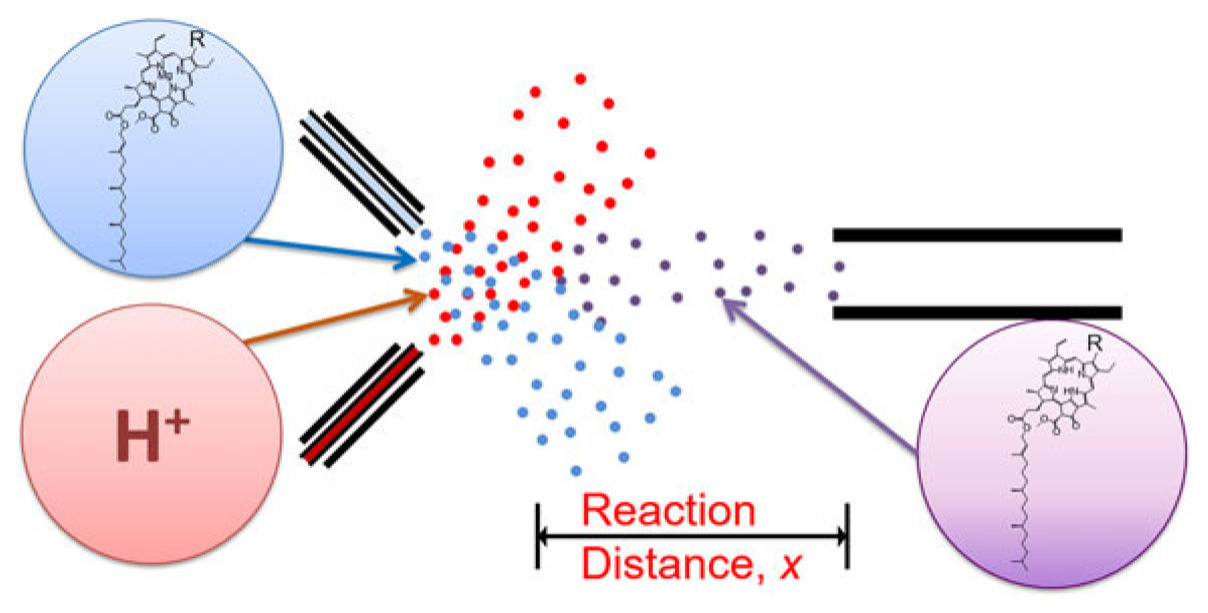
Experimental setup for chlorophyll demetallation kinetics using microdroplet fusion mass spectrometry.

The kinetics of chlorophyll *a* demetallation was recorded using microdroplet fusion mass spectrometry (Fig. 2). The two microdroplet streams containing chlorophyll *a* solution at 40 μM in methanol and aqueous HCl solution at concentrations that range from 0 to 35 mM were crossed to cause microdroplet fusion. The fused microdroplets traveled at a relatively constant speed of about 84 ± 18 m s^−1^ and entered into the heated capillary inlet of the mass spectrometer where reactions were stopped. The reaction time in the fused microdroplets at a mean size of 14 ± 6 μm was controlled by adjusting the traveling distance *x* between the droplet fusion region to the mass spectrometer inlet. For 3 μs temporal resolution, the distance *x* was increased by 250 μm using a manual micro-positioner. Mass spectra of chlorophyll *a* with HCl at the final concentration of 35 mM at different distance *x* were provided in Fig. 2. The signal intensity at *m*/*z* 893·53 corresponding to chlorophyll *a* (Chl *a*) decreased while the signal intensity at *m*/*z* 871·57 corresponding to phaeophytin (Phe) increased as the distance *x* increased. The reaction time was calculated by integrating the speed of fused droplet over distance *x* as provided in our previous work (Lee *et al.* 2015b).

The demetallation kinetics is first order in [Chl *a*] and second-order in [H^+^], so that

(1) $$d[\text{Chl}\;a]/dt=-k[\text{Chl}\;a]{\left[H^{+}\right]}^{2},$$

where *k* denotes the reaction rate constant, respectively. Because the H^+^ concentration is in great excess compared with the concentration of Chl *a*, we replace *k*[H*^+^*]^2^ by *k*_app_, and integrate Eq. (1) to yield the expression

(2) $${\left[\text{Chl}\;a\right]}_{t}={\left[\text{Chl}\;a\right]}_{0}\exp(-k_{\text{app}}t).$$

**Fig. 4:**
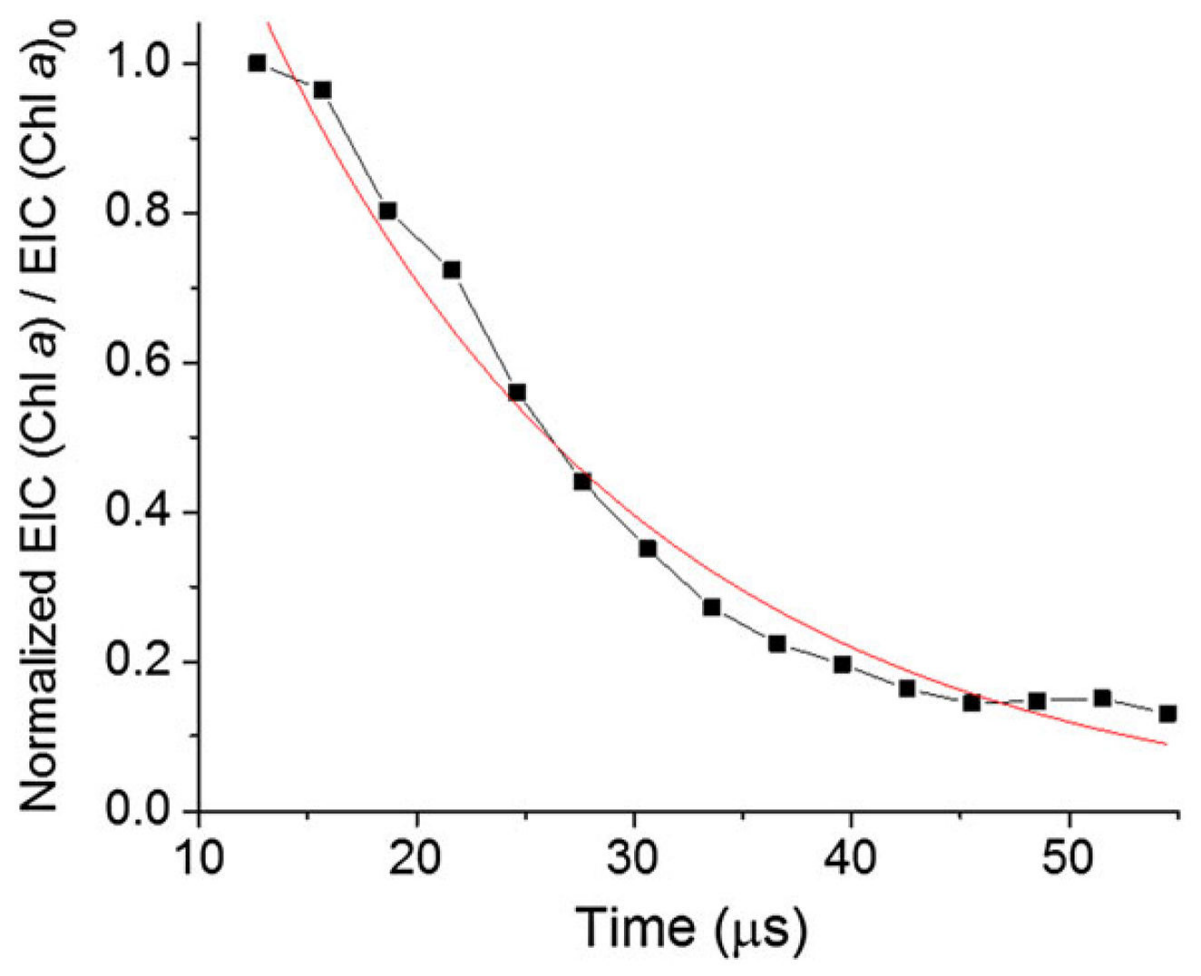
Kinetics of chlorophyll *a* demetallation recorded with microdroplet fusion mass spectrometry. The red line is the best fit to an exponential decay curve. EIC = extracted ion current.

In other words, the normalized signal intensity of chlorophyll *a* divided by the initial intensity of chlorophyll *a* follows a simple exponential decay function as a function of reaction time, which is the distance *x* divided by the microdroplet velocity (Fig. 4). The signal intensities at different distances *x* were normalized to the total ion current at each position to compensate for a signal loss caused by dispersion of fused microdroplets. The normalized chlorophyll *a* signal was fitted to an exponential decay curve shown by the red curve in Fig. 4. We expected evaporation of a fraction of solvent in the fused microdroplets, while they were traveling to the mass spectrometer inlet. However, a good fit to an exponential decay (*R*^2^ = 0·98) suggests that evaporation was minimal and played a negligible role in determining the kinetics.

**Fig. 5:**
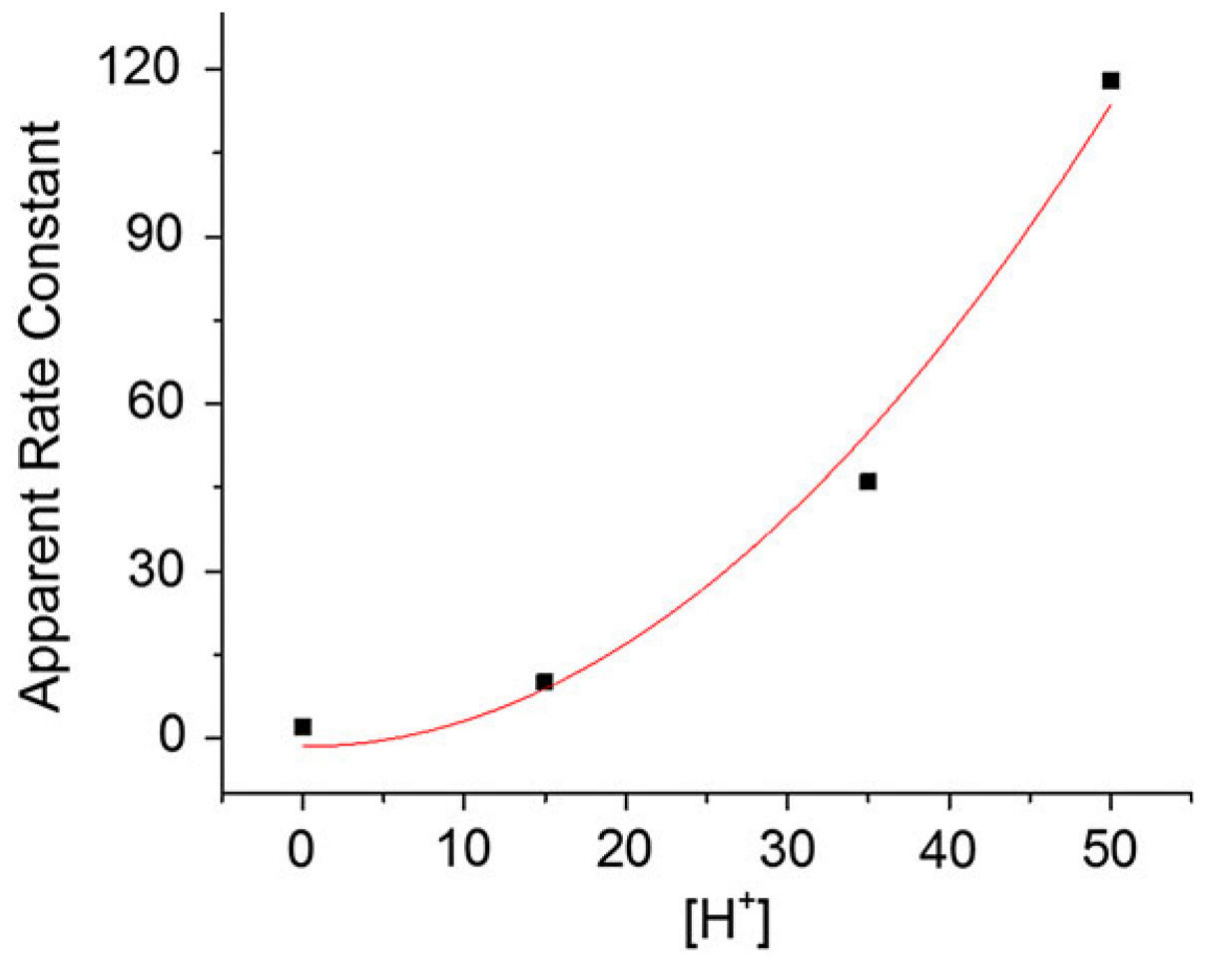
Apparent rates of acid-induced chlorophyll *a* demetallation at different concentrations of hydrogen ion. The red line is the best fit to a quadratic dependence on [H^+^].

The rate constant *k* of chlorophyll demetallation measured in microdroplets was 46 ± 6 mM^−2^ s^−1^. The reported rate constant measured in bulk solution is 0·048 ± 0·002 mM^−2^ s^−1^ (Wilson & Konermann, 2003). Thus, the reaction rate constant in microdroplets is estimated to be 960 ± 120 times greater than the one measured in bulk solution. The apparent rate constants of chlorophyll *a* demetallation *k*_app_ measured at different concentrations of H^+^ are depicted in Fig. 5. The data fit well a quadratic curve (*R*^2^ = 0·96), as expected. The approximately thousand-fold acceleration was found to be constant regardless of the H^+^ concentration in the microdroplets. It is noted that there was a sizable rate of demetalation in microdroplets, even when no HCl was added. This behavior is discussed below in terms of enhanced H^+^ concentration on the surface of the microdroplets.

### 3.2. Factors influencing reaction acceleration

**Fig. 6:**
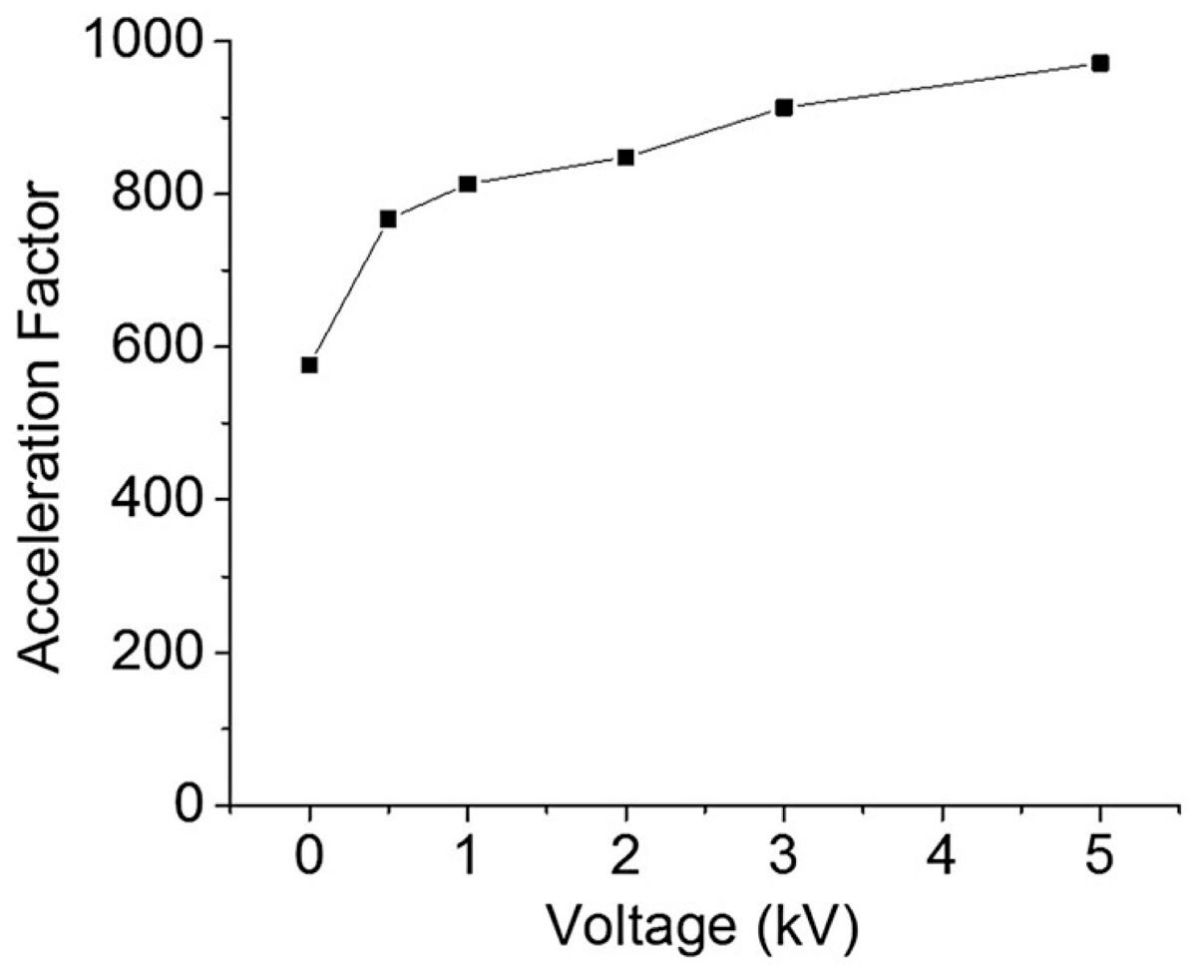
Effect of voltage applied to microdroplets on reaction rate acceleration factor compared with that in bulk solution.

Several candidates are thought to be responsible for the reaction rate acceleration in microdroplets. These include charge, localization of reactants at the interface, charge separation, micro-confinement, electric field and evaporation of solvent (Bain *et al.* 2016; Banerjee & Zare, 2015; Lee *et al.* 2015a; Li *et al.* 2016; Yan *et al.* 2016). We address the mechanism of this reaction acceleration by characterizing several factors among these candidates causing the reaction acceleration. First, we examined the effect of varying the voltage between 0 and 5 kV. The change in reaction rate acceleration in microdroplets compared to bulk solution is plotted as a function of the voltage in Fig. 6. The acceleration factor was about 600 without applying voltages to the microdroplets. The acceleration increased as the applied voltage increased. At 0·5 kV, the change in acceleration became almost saturated, meaning the introduced charge density at the surface of the microdroplets were almost saturated at the voltage as low as 0·5 kV. It should be noted that there was a considerate reaction acceleration occurring even without applying voltage to the microdroplets. This result suggests that a major portion of acceleration originates from other factors.

**Fig. 7:**
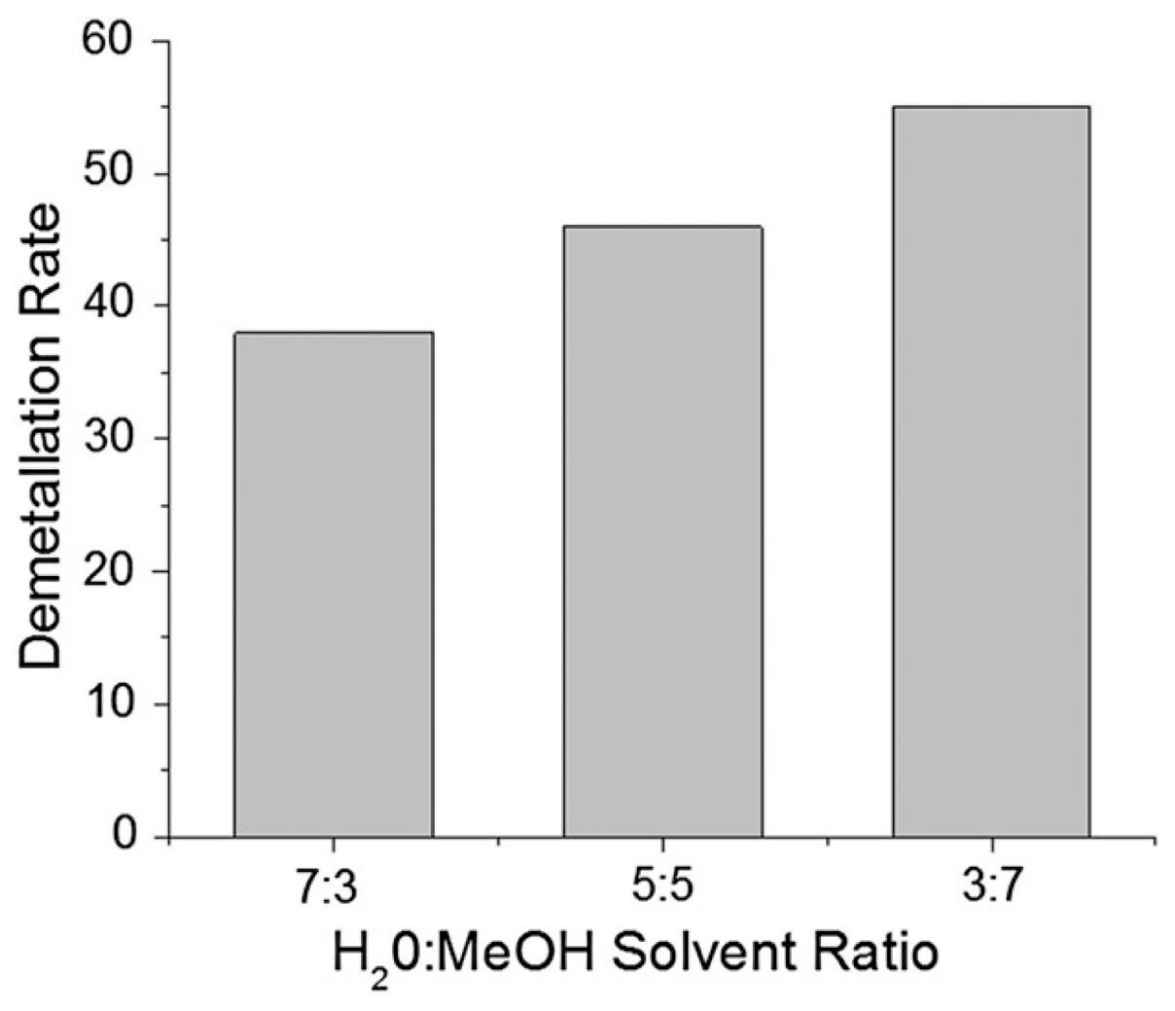
Effect of solvent composition on the demetallation rate of chlorophyll *a*. MeOH denotes methanol.

We then studied the effect of different solvent composition on the reaction acceleration. We measured demetallation rate constant in microdroplet with a different solvent mixing composition between water and methanol. The vapor pressure of water and methanol at 20 °C is about 17·5 and 98 torr. Therefore, the liquid in the microdroplets containing 70% methanol are expected to evaporate more rapidly than the microdroplets containing 30% methanol. The faster evaporation should lead to the increase of concentration of reactants, consequently, the increase of measured rate constant. The reaction rates increased by only about 20% as the percentage of methanol increased from 30 to 70% (Fig. 7). This behavior suggests that the influence of solvent evaporation was not significant.

### 3.3. Different kinetics between chlorophyll a and b

We compared the demetallation kinetics between chlorophyll *a* and *b* to assess the effect of different chemical structures on reaction acceleration in microdroplets. Figure 1*b* and c show the different chemical structures of chlorophyll *a* and *b*. A methyl group on the chlorin ring in chlorophyll *a* is replaced by a CHO group in chlorophyll *b*. Different side chains in chlorophylls exhibit different properties including absorption, redox potential, fluorescence, demetallation kinetics, etc. (Saga & Tamiaki, 2012). Chlorophyll *b* is more stable than chlorophyll *a* against proton attack (Hirai *et al.* 2009). Therefore, the demetallation kinetics of chlorophyll *b* is known to be slower than chlorophyll *a*.

The kinetics of chlorophyll *a* and *b* in microdroplets were measured using microdroplet fusion mass spectrometry under the identical conditions of 40 μM chlorophyll concentration, 35 mM HCl concentration, and an applied voltage of 5 kV. Table 1 summarizes the comparison between chlorophyll *a* and *b*. The measured rate constant of chlorophyll *b* demetallation was lower than chlorophyll *a*, which is consistent with the literatures (Hirai *et al.* 2009). However, the change in acceleration in microdroplets for chlorophyll *b* as a function of the H^+^ concentration was greater than chlorophyll *a*, suggesting that in the microdroplet environment chlorophyll *b* might be more susceptible to H^+^ attack.

## 4. Discussion and conclusions

We have studied the kinetics of chlorophyll demetallation occurring in microdroplets, and we found that the demetallation reaction rate is significantly faster in microdroplets that in bulk solution. We characterized the reaction rate acceleration in microdroplets by examining several factors influencing the acceleration. We investigated the effect of charge on the reaction rate acceleration by measuring the kinetics of chlorophyll demetallation under different voltages. Although the increased voltage from 0 to 5 kV increased the reaction acceleration factor, the major portion of the reaction acceleration was attributed to the other factors possibly, including the confinement effect and electric field at the microdroplet–air interface rather than the net charge introduced into the microdroplets. It should be noted that a significant reaction acceleration by approximately 600 times occurred with no voltage. It has been reported that neutral water exhibits a separation of the charge between hydronium ion and hydroxide ions distributing non-uniformly between the surface and inside of the water microdroplet (Vácha *et al.* 2007). Although the exact distribution of the autoionized ions and acidity at the air-water interface remain controversial (Beattie *et al.* 2009; Buch *et al.* 2007; Petersen & Saykally, 2008), it needs to be noted that the surface of the water may not be neutral. This unbalanced surface charge can still play a certain role for the reaction acceleration, for instance, by acid/base catalytic effect (Banerjee & Zare, 2015; Girod *et al.* 2011) or formation of electric field (Yang *et al.* 2011). These surface charge and catalytic effects are facilitated in microdroplets as the surface-to-volume ratio increases as size of the droplets decreases. We suggest that the large surface-to-volume ratio of microdroplets compared with the same total volume in a bulk solution is a major factor in causing reaction acceleration.

We also examined the influence of different solvent composition by varying the mixing ratio between water and methanol. The different solvent composition affected the reaction rate of chlorophyll *a* demetallation only by 20%. This observation demonstrates that the solvent composition does not significantly affect the acceleration. Given about four times higher evaporation rate of methanol compared to water at room temperature, the reaction rates in the microdroplets containing a higher portion of methanol are expected to be accelerated compared with water. However, the reaction rates differ between the microdroplets containing 30 and 70% methanol by only 20%. This implies that evaporation does not dominate in causing acceleration of this reaction in microdroplets.

It might be wondered why the acid-induce demetallation of chlorophyll *a* and chlorophyll *b* differed in the microdroplet compared with bulk solution. Several factors might contribute to the behavior, such as different surface adsorption and different effects of electric field at the air-liquid interface. Chlorophyll *b* possessing a –CHO group is more hydrophilic than chlorophyll *a* possessing a –CH_3_ group. We speculate that the differences in the hydrophilicity between chlorophyll *a* and *b* might cause the differences in the acceleration factor by affecting the surface adsorption. The surface catalytic effect of liquid microdroplet surface can be also influenced by the different polarity of the different groups between chlorophyll *a* and *b*. The electric field may also play a role in accounting for the difference as the electric field can influence the orientation of reactant molecules during reactions.

In the present study, the accelerated kinetics of chlorophyll demetallation suggests the reaction acceleration was mainly attributed to the micro-confinement effect rather than the introduced charges, solvent composition, and solvent evaporation effect in microdroplet. The behavior of accelerated reaction rate in biological reactions in the present work illustrates that reactions of biomolecules in a confined environment similar to the cell may behave differently than what has been reported through assays using bulk solutions.

## Figures and Tables

**Fig. 3:**
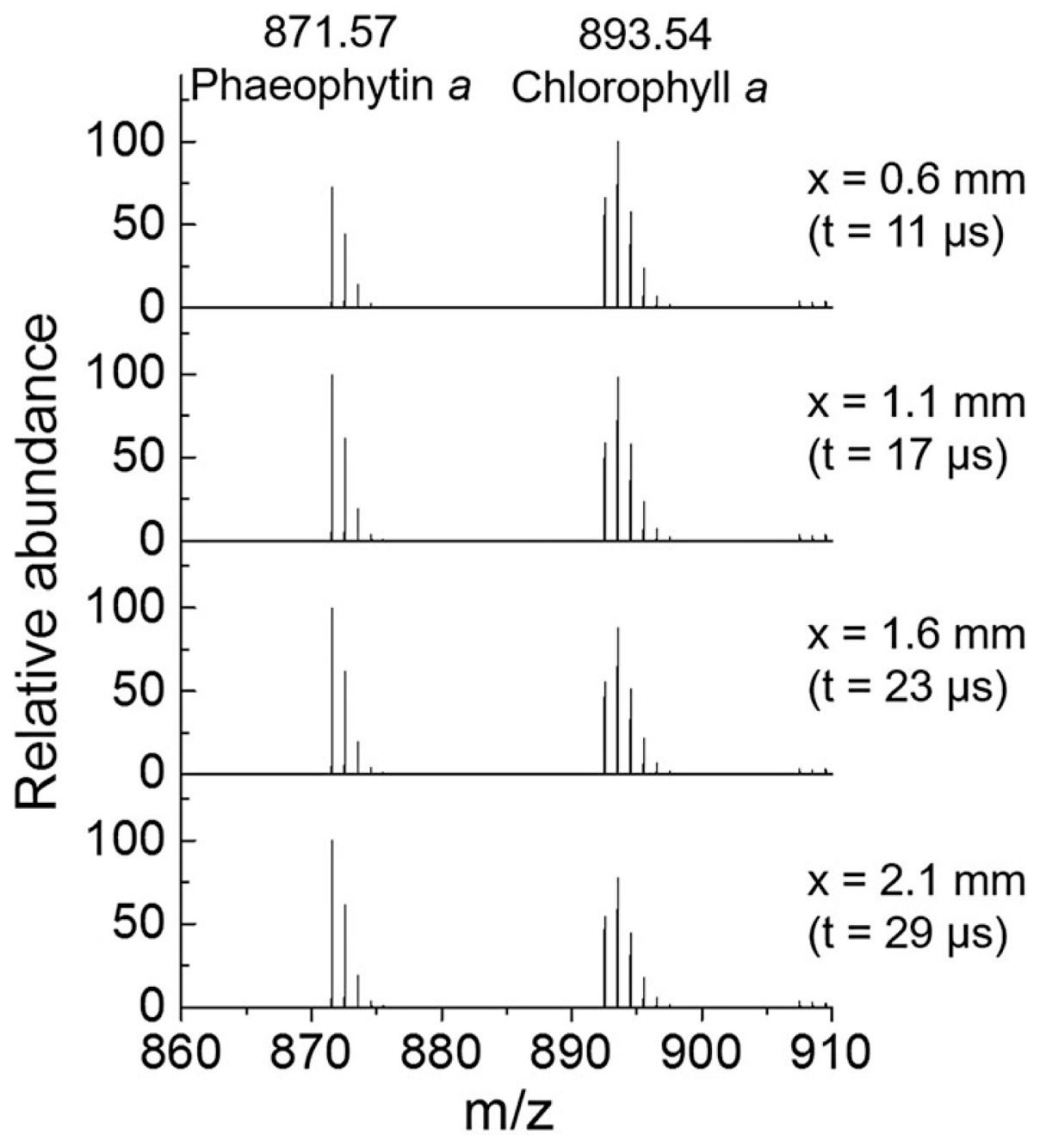
Mass spectra of chlorophyll *a* at different traveling distances of fused microdroplets. The intensity of chlorophyll *a* decreases, whereas the intensity of phaeophytin *a* increases as the traveling distance *x*, as defined in Fig. 2, and the corresponding reaction time *t* in the fused microdroplets increase.

**Table 1:** Comparison of demetallation rates between chlorophyll a and b in microdroplets. The kinetic measurements were conducted in fused water:methanol (1:1, v/v) microdroplets containing 35 mM HCl and 40 μM chlorophylls under 5 kV spray condition

	Chlorophyll *a*	Chlorophyll *b*
Rate constant in bulk solution (mM^−2^ s^−1^)	0·048^a^	0·0018^b^
Rate constant in microdroplets (mM^−2^ s^−1^)	46	8·8
Acceleration factor	960	4900

a Data taken from (Wilson & Konermann, 2003).

b Data taken from (Gerola *et al.* 2011; Mazaki *et al.* 1992).
